# *Acinetobacter baumannii* up-regulates LncRNA-GAS5 and promotes the degradation of STX17 by blocking the activation of YY1

**DOI:** 10.1080/21505594.2021.1953851

**Published:** 2021-07-25

**Authors:** Zhiyuan An, Wenyi Ding

**Affiliations:** a Medical Research Center, Beijing Chaoyang Hospital, Capital Medical University, Beijing, China; b Department of Clinical Laboratory, Peking Union Medical College Hospital, Chinese Academy of Medical Sciences, Beijing, China

**Keywords:** *Acinetobacter baumannii*, lncRNA-GAS5, yin-yang 1, synapsin 17, autophagy, IFN-β

## Abstract

*Acinetobacter baumanniitriggers* autophagy, affects the degradation of autophagy, and causes severe inflammatory injury. LncRNA growth arrest-specific transcript 5 (LncRNA-GAS5) and Yin and Yang 1 (YY1) are known to play an important role in the regulation of autophagy, however, the precise role of LncRNA-GAS5 and YY1 in the damage to autophagy caused by *Acinetobacter baumanniiremains* unclear. The aim of this study was to investigate the role of LncRNA-GAS5 and YY1 in the regulation of autophagy induced by *Acinetobacter baumannii*. We found that LncRNA-GAS5 was up-regulated following infection with *Acinetobacter baumannii*, thus resulting in the degradation of STX17, autophagy disorders, and the aggravated replication of *Acinetobacter baumannii*. We also analyzed the mechanism of interaction between LncRNA-GAS5 and YY1 and found that YY1 regulated its expression in a negative manner by binding to the promoter of LncRNA-GAS5. LncRNA-GAS5 and YY1 had opposite effects on the expression of STX17, this process maintained the stable expression of STX17. Following *Acinetobacter baumannii* infection, YY1 was down regulated and then separated from the binding region of LncRNA-GAS5, thus resulting in the activation of LncRNA-GAS5 transcription and reduction in STX17 protein expression. Finally, we infected LncRNA-GAS5 knockdown mice with *Acinetobacter baumannii*, the expression levels of IFN-β in the lungs increased significantly, this alleviated lung injury. In conclusion, our work demonstrated the mechanism by which *Acinetobacter baumannii* infection can cause the degradation of STX17. We also demonstrated that LncRNA-GAS5 may be a potential therapeutic target for the treatment of lung injury induced by *Acinetobacter baumannii*.

## Introduction

*Acinetobacter baumannii* is an opportunistic nosocomial pathogen that causes hospital-acquired pneumonia, septicemia, and urinary tract infection, especially in critically ill patients [1,2]. Over recent years, the excessive use of antibiotics has led to the emergence of a highly drug-resistant form of *Acinetobacter baumannii*, this has become one of the most difficult pathogens to treat in the clinic [3]. The pathogenesis of *Acinetobacter baumannii* remains unclear and the effect of antibiotic treatment is not satisfactory. Therefore, it is vital that we study the mechanisms by which *Acinetobacter baumannii* affects the host immune system and how the host immune system counteracts such infection.

Autophagy is a process in which autophagosomes fuse with lysosomes to cause the degradation of inclusion bodies. Autophagy also plays an important role in host resistance to pathogen infection [4]. However, bacteria have evolved the ability to survive in autophagosomes, control the formation of autophagosome, and interfere with the degradation of autophagy. Autophagosomes have become a natural protective cover for bacterial replication [5]. Very few studies have investigated the interaction between *Acinetobacter baumannii* and autophagy. In 2014, Rumbo carried out research on *Acinetobacter baumannii* and reported that outer membrane protein 33 (Omp33) mediates the autophagy of HEp2 cells and leads to a failure of autophagy degradation, thereby enhancing bacterial replication [6]. In another study, Wang et al. found that *Acinetobacter baumannii* promotes cell autophagy and that this has a positive effect on clearing bacterial infection [7]. In our previous research, we revealed that *Acinetobacter baumannii* outer membrane protein A (OmpA) inhibited the degradation of p62/STSQM1, blocked the formation of autolysosomes, and led to IL-1β release and inflammatory cell death [8]. STX17, also known as synapsin 17, is a vesicular protein located on the surface membrane of mature autophagosomes. STX17 plays a role in the process of autophagy by controlling fusion of the autophagosome membrane with the lysosome membrane, therefore, STX17 is the key protein that controls the formation of autophagosomes and lysosomes [9]. Numerous studies have shown that pathogens evade autophagic degradation by inhibiting the formation of autophagosomes and lysosomes by degrading STX17 [10,11]. However, whether this mechanism is involved in the suppressive effect of *Acinetobacter baumannii* on autolysosome formation remains unclear.

Long non-coding RNAs (LncRNAs) have many functional activities and are involved in a wide range of biological and disease processes. LncRNAs can regulate gene expression in many ways, including transcriptional regulation, post-transcriptional regulation, and epigenetic regulation [12,13]. Studies have shown that via these mechanisms, LncRNA can regulate autophagy and control bacterial infection [14,15]. However, the specific role of LncRNAs in the process of *Acinetobacter baumannii* infection has yet to be studied. LncRNA-GAS5, also known as growth arrest-specific transcript 5, acts as a tumor suppressor and plays an important role in tumor proliferation, invasion, and apoptosis [16,17]. GAS5 is not only involved in the biological aspects of tumors, it also plays a key role in regulating autophagy. It has been reported that GAS5 can regulate autophagy-related genes and alter levels of autophagy by sponging microRNAs. So far, the regulatory effects of GAS5 on autophagy have been generally based on this mechanism [18,19]. At present, little is known about the specific role of GAS5 in bacterial and viral infection. In a previous study, Qian et al found that GAS5 binds to the NS3 protein of hepatitis C virus and interferes with its activity to inhibit viral replication [20]. Other studies have shown that GAS5 inhibited HIV-1 replication by binding to miR-873 [21]. These results suggest that GAS5 plays a key role in regulating autophagy and controlling the replication of pathogens.

Yin and Yang 1 (YY1) is a highly conserved zinc-finger transcription factor that binds directly or indirectly to a large number of gene promoters to activate or repress expression *via* chromatin remodeling or histone modification [22]. Studies have shown that YY1 plays an important role in the regulation of tumorigenesis and progression, apoptosis, and autophagy [23]. Du et al. reported that YY1 and TFEB cooperated to control the fusion of autophagosomes and lysosomes [24]. In another study, Yang et al. found that YY1 and miR-30a regulate autophagy via negative feedback [25]. In addition, YY1 is also known to interact with LncRNA. For example, YY1 binds directly to the LncRNA-PVT1 promoter and activates its transcription to promote the proliferation and invasion of lung cancer cells [26]. Moreover, YY1 activates LncRNA-ARAP1-AS1 to activate the Wnt/β-catenin signaling pathway and promote the metastasis and proliferation of colon cancer cells [27]. Recently, Zhang et al. found that the complex created by YY1 and GAS5 regulates PFKFB3 to initiate cerebral ischemia-reperfusion injury and that the knockout of LncRNA-GAS5 protects against ischemic brain injury and improves neurological function in mice [28]. However, we do not know if *Acinetobacter baumannii* affects the functional activity of YY1. Moreover, the functions and mechanisms involving cross-talk between GAS5 and YY1 in the induction of autophagy by infection with *Acinetobacter baumannii* have yet to be elucidated.

In this study, we investigated the molecular mechanisms underlying the action of *Acinetobacter baumannii* on STX17-induced autophagy degradation disorder. Our research indicated that the GAS5/YY1/STX17 pathway may represent a new regulatory network that controls the disruption of autophagy in response to infection by *Acinetobacter baumannii*.

## Materials and methods

### Acinetobacter baumannii *strains and infection*

We obtained the *Acinetobacter baumannii* strain 19606 from the American Type Culture Collection (ATCC). *Acinetobacter baumannii* cultures were then grown on blood plates for 24 h. Precipitates were then harvested by centrifugation and resuspended in a broth medium. HeLa cells (ATCC®CCL-2) were then infected with *Acinetobacter baumannii* strain 19606 at an Multiplicity of infection (MOI) of 10.

## Mouse models

All mice were housed in a specific-pathogen-free (SPF) environment at the Medical Research Center of Beijing Chaoyang Hospital. We used 6–8-week-old C57BL/6 male mice for experiments. Firstly, the trachea of mice was exposed surgically. Then, purified adeno-associated viral vector serotype (AAV) expressing the GAS5 gene was injected directly into the trachea at a concentration of 1.3 × 10^12^ virus particles/mouse. After 3 weeks, this procedure led to the generation of LncRNA-GAS5 knockdown mice. Sham and *Acinetobacter baumannii* infection groups were then maintained for 3 weeks (post-AAV-null or AAV-GAS5 injection).The mice were then infected with 6.4x10^5^Colony-Forming Unit (CFU) of *Acinetobacter baumannii* by intraperitoneal injection.

## Cell culture

Human cervical cancer epithelial cells (HeLa cells, ATCC®CCL-2, American Type Culture Collection) were cultured in Dulbecco’s modified Eagle’s medium (Gibco, Grand Island, NY, USA) supplemented with 10% fetal bovine serum and 100 U/ml penicillin/streptomycin in a humidified incubator containing 5% CO_2_at 37°C.

## Plasmid construction and cell transfection

pcDNA3.1-GAS5, pcDNA3.1-YY1, and pcDNA3.1-STX17 plasmids were constructed by Synbio Tech Co. Ltd (Suzhou, China). The target sequence of lncRNA-GAS5 shRNA and STX17 siRNA (at a final concentration of 75pmol) were constructed by Synbio Tech Co. Ltd. YY1 shRNA was constructed by Genepharma (Shanghai, China).The knock-down sequences used are listed in Table S1. The pRL-TK Renilla plasmid was purchased from Promega (Madison, WI, USA). All plasmids were transfected using Lipofectamine 3000 Transfection Reagent (Invitrogen, Carlsbad, CA, USA) according to the manufacturer’s protocol.

## RNA extraction and mRNA quantification by real-time PCR

Cells were seeded in six-well plates and cultured. Total RNAs were extracted from cultured cells using TRIzol reagent (Invitrogen, Carlsbad, CA, USA) according to the manufacturer’s protocol. First-strand cDNA was generated using the First-Strand cDNA Synthesis SuperMix (TransGen Biotech Co. Ltd) and Green miRNA Two-Step qRT-PCR SuperMix (TransGen Biotech co. Ltd) for quantitative real-time PCR, according to the manufacturer’s instructions. GAPDH was used as an endogenous control. The region specific primers used are listed in Table S1.All experiments were repeated three times independently.

## Western blotting

Cell lysates were prepared by using 4 X SDS (Sodium dodecyl sulfate) sample buffer. Proteins were then loaded onto 10 or 15% SDS-PAGE gels and transferred onto polyvinylidene fluoride (PVDF) membranes (Millipore, USA). Membranes were then incubated with a specific antibodies against STX17(1:1000, sigma, HPA001204), STX17 (1:1000, Abcam, ab229646), LC3A/B(1:500, Cell Signaling Technology, #12741), SQSTM1/p62(1:1000, Abcam, ab56416), GAPDH(1:1000, Cell Signaling Technology, #5174), ATG14 (1:200, Cell Signaling Technology, #96752), p-ULK (1:1000, Cell Signaling Technology, #14202), YY1 (1:1000, Cell Signaling Technology, #46395), and YY1(1:500, Proteintech, 66281-1-Ig). Band intensity was then quantified by Image J software (NIH, Bethesda, MD, USA).

## Dual-luciferase reporter assays

The YY1 binding site in the LncRNA-GAS5 and STX17 promoter regions (2.0 kb sequence upstream of the transcription initiation site, TSS) were amplified by PCR and cloned downstream of the pGL3 basic luciferase reporter plasmid, thus allowing us to generate pGL3-STX17 and pGL3-GAS5 plasmids. For reporter assays, HeLa cells were co-transfected with the indicated luciferase reporter vector and pRL-TK Renilla luciferase plasmids in a 24-well plate. Luciferase activity was measured 24 h after transfection using the Dual-Luciferase Reporter Assay System following the manufacturer’s protocol (Promega, E1960, Madison, WI, USA).All experiments were repeated three times independently.

## *In vitro* invasion assay

HeLa cells were seeded at a concentration of 1x10^4^cells per well in a 24 well plate. After 24 h of incubation, cells were infected with *Acinetobacter baumannii* for 24 h using an MOI of 10. Subsequently, cells were washed three times with DuIbecco’s modified eagIe’s medium (DMEM) medium and incubated with DMEM medium containing 10% FBS and 250 μg/ml gentamicin for 1.5 hours to kill extracellular *Acinetobacter baumannii*. Then, the cells were washed three times with sterile PBS, centrifuged at 1000rpm for 3 min and the pellet lysed using 200 μl of 0.1% Triton X-100 for 10 min at room temperature. Then, 100 μl was plated (in triplicate) on LB agar and the CFU was determined after overnight cultivation.

## Florescence *in situ* hybridization and immunofluorescence microscopy

HeLa cells were fixed with 4% paraformaldehyde for 15 min. Next, cells were permeabilized with 0.3% Triton X100 for 10 min. Then, the cells were incubated with a LncRNA-GAS5-specific probe (Genepharma, Suzhou, China) at 37°C overnight. The nucleus was stained with DAPI and images were acquired using an Olympus X51 fluorescence microscope (objective, 40X).

Immunofluorescence Assay (IFA) was performed after Fluorescence in situ hybridization (FISH) or independently. HeLa cells were fixed with 4% paraformaldehyde for 15 min and then permeabilized with 0.3% Triton X100 for 10 min. A LC3B or YY1 monoclonal antibody was then incubated with the cells at 4°C overnight. Cells were then washed with PBS three times, mixed with an appropriate fluorescent secondary antibody, and incubated at room temperature for 1 hour. The nuclei were stained with DAPI and images were acquired with an Olympus X51 fluorescence microscope.

## RNA immunoprecipitation (RIP) assay

RNA immunoprecipitation (RIP) was performed using the EZ-Magna RIP Kit (Millipore, Burlington, MA, United States) in accordance with the manufacturer’s protocol. In brief, lysis buffer was used to dissolve HeLa cells to prepare a lysate. A monoclonal antibody against YY1 was then added to the lysate and then co-precipitated with protein A/G magnetic beads overnight. Finally, the purified RNA was detected by qPCR.

## Chromatin immunoprecipitation (ChIP) assay

ChIP assays were performed using the Simple ChIP Enzymatic Chromatin IP Kit, (Cell Signaling, #9002) in accordance with the manufacturer’s guidelines. Chromatin was incubated overnight with YY1 antibody (Cell Signaling, #46395) or control antibody (Cell Signaling, #2729) at 4°C with rotation and incubated with ChIP-grade protein G agarose beads for 2 h. The bound DNA was then analyzed by real-time PCR using primer sequences spanning the STX17 and GAS5 promoter. The region-specific primers used for these assays are listed in Table S1.

## Flow cytometry assay

Spleens were extracted from experimental mice was and ground with a 100 copper mesh in a 35 cm dish containing 2 ml of medium to prepare a suspension of spleen cells. After centrifugation at 1500 rpm for 5 min, the supernatant was discarded. Red blood cells were then lysed with red blood cell lysate for 5 minutes, centrifuged for 5 minutes, and the cell precipitation was suspended in cell staining buffer. Cells were counted and 1 × 10^5^ cells/100 µl were taken. The cells were incubated at room temperature for 20 minutes with 5 µl of APC-CD11b and PE-F4/80 antibody (BioLegend, San Diego, CA, USA). Next, the cells were washed twice with PBS and then resuspended with 500 µl of PBS. The number of macrophages was then detected by flow cytometry assay.

## Statistical analysis

Statistical analyses were performed using a two-tailed Student’s *t*-test. Data was presented as the mean and standard error of the mean [SEM]). Significant differences were assigned to p values<0.05, <0.01 (denoted by *, and **, respectively).

## Results

### *Levels of lncRNA-GAS5 were increased in HeLa cells after infection with* Acinetobacter baumannii

**Figure 1.**
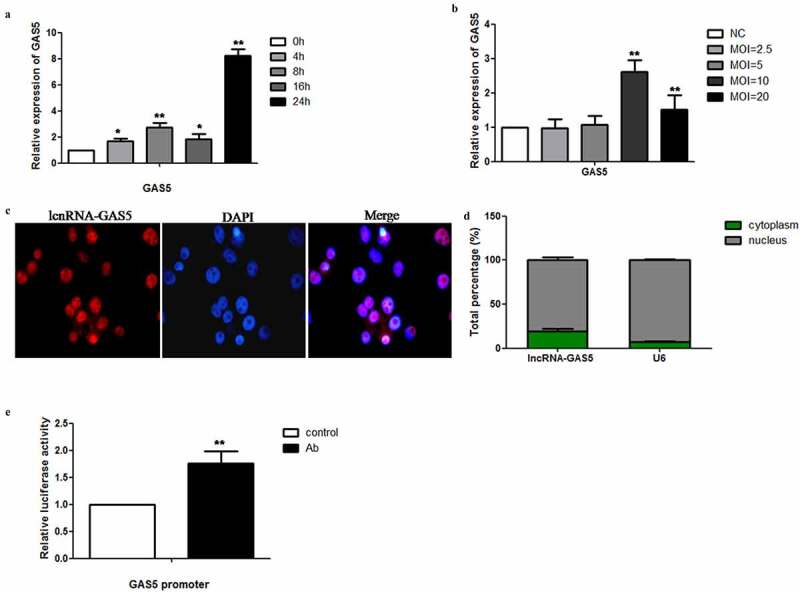
**LncRNA-GAS5 is increased in HeLa cells after *Acinetobacter baumannii infection***. 1A: The expression of GAS5 in HeLa cells infected with *Acinetobacter baumannii* (MOI = 10) at indicated time points was detected by qPCR. 1B: The expression of GAS5 in HeLa cells infected with *Acinetobacter baumannii* at indicated MOI for 24 hours by qPCR. 1C: The expression and subcelluar location of GAS5 in HeLa cells was determined by Fluorescence in situ hybridization analysis. DAPI-stained nuclei were blue. (Magnification X40). 1D: Percentage distribution of GAS5 in the nucleus and cytoplasm of HeLa cells. 1E: HeLa cells were infected with *Acinetobacter baumannii* at 24 hours or not infected, the activity of GAS5 promoter was detected by Dual-luciferase reporter assay

To explore the effect of *Acinetobacter baumannii* on LncRNA-GAS5 expression, we used real-time PCR to detect the expression of GAS5 in HeLa cells infected with *Acinetobacter baumannii* at different times and concentrations. We found that the expression levels of GAS5 increased with the increased *Acinetobacter baumannii* infection time, reaching a peak at 24 hours (Figure 1a). In addition, we found that HeLa cells were infected with MOI = 2.5 or 5, the expression level of LncRNA-GAS5 did not increase significantly compared with the uninfected group, However, when HeLa cells were infected with MOI = 10, the expression level of LncRNA-GAS5 reached the highest level. After infection with MOI = 20, the expression of LncRNA-GAS5 decreased. (Figure 1b). The localization of LncRNA is closely related to its function, therefore, we used fluorescence *in situ* hybridization (FISH) analysis to detect the subcellular localization of LncRNA-GAS5 in HeLa cells. We found that GAS5 was mainly localized in the nucleus of HeLa cells (Figure 1c-d). Moreover, Dual-luciferase reporter assays showed that the activity of the GAS5 promoter was significantly enhanced by *Acinetobacter baumannii* (Figure 1e).

All data were presented as means ± SEM of three independent experiments, Values were significant at *p < 0.05, **p < 0.01 as indicated.

### Acinetobacter baumannii *increased the transcription of LncRNA-GAS5 in a YY1-dependent manner*

**Figure 2.**
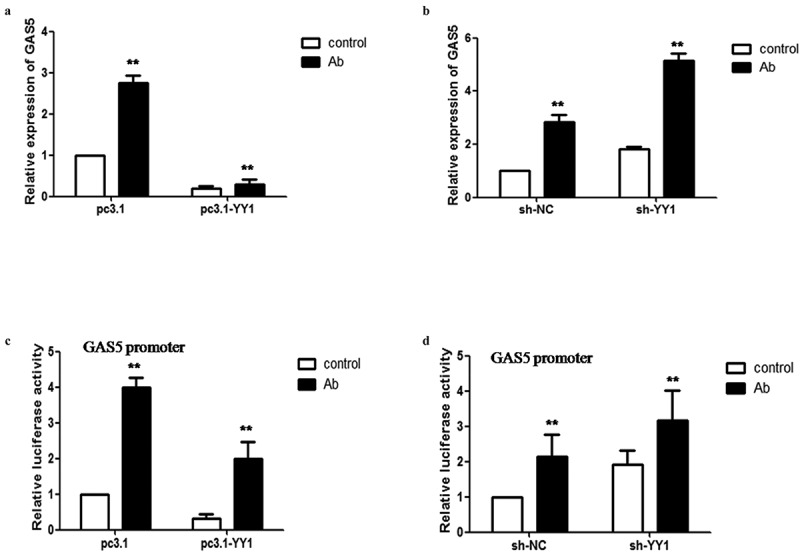
**Transcription factor YY1 regulates the transcription of LncRNA-GAS5 mediated by *Acinetobacter baumannii***. 2A: HeLa cells transfected with pc3.1-YY1 or pcDNA3.1 were infected with *Acinetobacter baumannii* for 24 hours, the expression of GAS5 levels were analyzed with qPCR. 2B: HeLa cells transfected with sh-YY1 and sh-NC were infected with *Acinetobacter baumannii* for 24 hours, the expression of GAS5 levels were analyzed with qPCR. 2C: Dual- luciferase reporter assay of GAS5 promoter activity in YY1-overexpressing cells after *Acinetobacter baumannii* for 24 hours. 2D: Dual- luciferase reporter assay of GAS5 promoter activity in YY1-knockdown cells after *Acinetobacter baumannii* for 48 hours

Next, we considered whether the increase of GAS5 induced by *Acinetobacter baumannii* was mediated by YY1. We overexpressed YY1 in cells and then stimulated the cells with *Acinetobacter baumannii* for 24 hours. We found that the overexpression of YY1 significantly attenuated GAS5 expression induced by *Acinetobacter baumannii* infection (Figure 2a). In contrast, the knockdown of YY1 expression with sh-YY1 significantly increased the expression levels of GAS5 induced by *Acinetobacter baumannii* infection (Figure 2b). Moreover, Dual-luciferase reporter assays showed that the overexpression of YY1 inhibited the activation of the GAS5 promoter after *Acinetobacter baumannii* infection, while the knockdown of YY1 promoted activation of the GAS5 promoter by *Acinetobacter baumannii* (Figure 2c-d). These results indicated that YY1 is required for GAS5 transcription in response to *Acinetobacter baumannii* infection.

All data were presented as means ± SEM of three independent experiments, Values were significant at **p < 0.01 as indicated.

## LncRNA-GAS5 promoted autophagy but inhibited the degradation of autophagy

**Figure 3.**
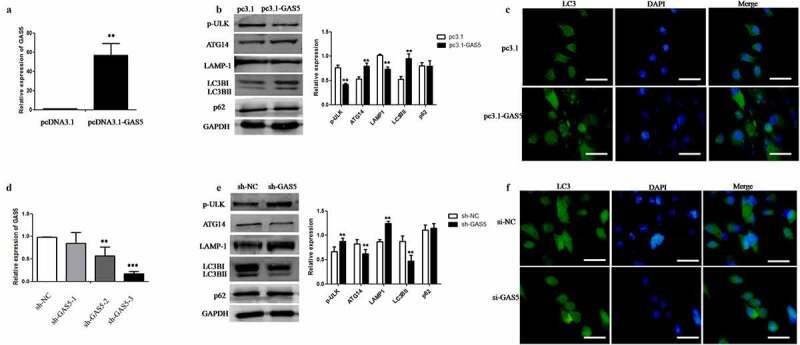
**LncRNA-GAS5 promotes autophagy but inhibits autophagy degradation**. 3A: After transfection of pcDNA3.1 or pc3.1-GAS5 plasmid into HeLa cells respectively for 24 hours, the expression level of GAS5 was detected by qPCR. 3B: The expression level of autophagy related proteins in HeLa cells transfected with pc3.1-GAS5 plasmid was detected by Western blot assay. 3C: The expression level of LC3B in HeLa cells transfected with pcDNA3.1 or pc3.1-GAS5 plasmid was detected by immunofluorescence microscopy. The nuclei were stained with DAPI (blue). Scale bar: 10 μm. 3D: After transfection of sh-GAS5 plasmid into HeLa cells for 48 hours, the expression level of GAS5 was detected by qPCR. 3E: The expression level of autophagy related proteins in HeLa cells transfected with sh-NC or sh-GAS5 plasmid was detected by Western blot assay. 3F: The expression level of LC3B in HeLa cells transfected with si-NC or si-GAS5 plasmid was detected by immunofluorescence microscopy. The nuclei were stained with DAPI (blue). Scale Bar: 10 μm

To investigate the function of GAS5 in autophagic degradation, we enhanced the levels of GAS5 in HeLa cells by transfection with pcDNA3.1-GAS5. We found that the expression of GAS5 was significantly higher after pc3.1-GAS5 transfection (Figure 3a). Furthermore, we analyzed the effect of GAS5 overexpression on the expression of autophagy-related proteins in HeLa cells. Western blot analysis of autophagy-related proteins showed that levels of p-ULK (ser757) had decreased, but the levels of ATG14 and LC3B had increased, thus indicating that GAS5 activated autophagy. In addition, GAS5 reduced the expression of LAMP1 and did not change the levels of p62 protein, thus suggesting that GAS5 may interfere with autophagic degradation (Figure 3b). Immunofluorescence staining further showed that GAS5 could increase the expression of LC3B (Figure 3c). In contrast, we designed three independent short hairpin RNAs for human GAS5 (sh-GAS5) to knockdown the expression of GAS5 in HeLa cells. qPCR assays showed that the expression of GAS5 was significantly downregulated by sh-GAS5-3 (Figure 3d). Furthermore, western blot analysis showed that the expression of autophagy-related proteins had the opposite effects to the overexpression of GAS5 (Figure 3e). Immunofluorescence staining showed that the expression of LC3B decreased after the knockdown of GAS5 (Figure 3f). These results suggested that although GAS5 activates autophagy, it can also interfere with autophagy degradation. Furthermore, the knockdown of GAS5 attenuated autophagy but was also beneficial to autophagy degradation.

All data were presented as means ± SEM of three independent experiments, Values were significant at **p < 0.01 as indicated.

## LncRNA-GAS5 down-regulated STX17 to augment the intracellular survival of *Acinetobacter baumannii* in HeLa cells

**Figure 4.**
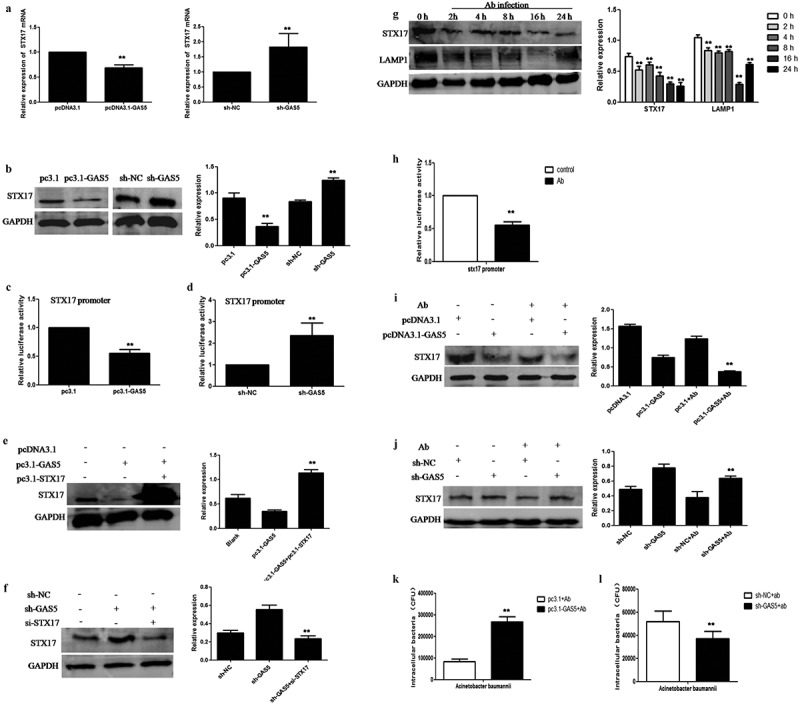
**LncRNA-GAS5 down-regulates STX17 to augments intracellular *Acinetobacter baumannii* survival in HeLa cells**. 4A: The expression level of STX17 mRNA was detected in HeLa cells by qPCR after transfected with pcDNA3.1 or pc3.1-GAS5 as well as sh-NC or sh-GAS5 plasmid respectively. 4B: The expression level of STX17 protein was detected by Western blot assay after transfected with pcDNA3.1 or pc3.1-GAS5 as well as sh-NC or sh-GAS5 plasmid respectively. 4C: Dual-luciferase reporter assays of STX17 promoter activity in HeLa cells which were transfected with pcDNA3.1 or pc3.1-GAS5 plasmids respectively. 4D: Dual-luciferase reporter assays of STX17 promoter activity in HeLa cells which were transfected with sh-NC or sh-GAS5 plasmids respectively. 4E: The expression of STX17 was detected by Western blot assay after transfected with pc3.1-GAS5 or co-transfection of pc3.1-GAS5 and pc3.1-STX17 plasmids respectively. 4F: The expression of STX17 was detected by Western blot assay after transfected with sh-GAS5 or co-transfection of sh-GAS5 and si-STX17 respectively. 4G: The expression levels of STX17 and LAMP1 in HeLa cells subjected to *Acinetobacter baumannii* infection for different times were assessed by Western blot assay. 4H: Dual-luciferase reporter assay was used to detect the activity of STX17 promoter after *Acinetobacter baumannii* infection for 24 hours. 4I-J: The plasmids of pcDNA3.1or pc3.1-GAS5 as well as sh-NC or sh-GAS5 were transfected respectively for 24 hours, and then infected with *Acinetobacter baumannii* for 24 hours. The expression of STX17 was detected by Western blot assay. 4K-L: The effect of GAS5 on the invasion of *Acinetobacter baumannii* into HeLa cells was detected by Gentamicin protection test

Since GAS5 affects the degradation of autophagy, STX17 represents the key protein with which to control autophagy degradation. We hypothesized that STX17 might be a potential target for GAS5. Coincidentally, the overexpression or knockdown of GAS5 significantly repressed or activated STX17 mRNA and protein expression in HeLa cells (Figure 4a-b). In addition, Dual-luciferase reporter assays showed that GAS5 significantly inhibited the STX17 promoter, while the knockdown of GAS5 significantly activated the STX17 promoter (Figure 4c-d). Moreover, we performed rescue experiments and found that the overexpression of STX17 could rescue the inhibitory effect of pcDNA3.1-GAS5 overexpression on STX17. Furthermore, siRNA against STX17 (si-STX17) was shown to rescue the activation effect of sh-GAS5 knockdown on STX17 (Figure 4e-f). Next, we investigated the effect of *Acinetobacter baumannii* on the expression of STX17 and LAMP1. Western blot analysis showed that *Acinetobacter baumannii* attenuated STX17 and LAMP1 expression in a time-dependent manner (Figure 4g). Dual-luciferase reporter assays showed that *Acinetobacter baumannii* inhibited the STX17 promoter (Figure 4h). To identify the effect of GAS5 on the expression of STX17 protein induced by *Acinetobacter baumannii* infection, we transfected pc3.1-GAS5 and GAS5-targeting shRNA into HeLa cells and infected the cells with *Acinetobacter baumannii*, then we determined the protein levels of STX17. We found that after the overexpression or knockdown of GAS5, the expression level of STX17 protein was decreased or increased more significantly than that in the *Acinetobacter baumannii* infection group. This suggests that GAS5 plays a key role in the degradation of STX17 protein induced by *Acinetobacter baumannii* (Figure 4i-j). Finally, we evaluated the effect of GAS5 on *Acinetobacter baumannii* replication and found that intracellular *Acinetobacter baumannii* growth was markedly increased in cells transfected with GAS5. In contrast, the growth of *Acinetobacter baumannii* was inhibited after GAS5 knockdown (Figure 4k-l). These results indicated that the knockdown of GAS5 reversed the degradation of STX17 induced by *Acinetobacter baumannii* and inhibited the infection and proliferation of *Acinetobacter baumannii* in HeLa cells.

All data were presented as means ± SEM of three independent experiments, Values were significant at **p < 0.01 as indicated.

## LncRNA-GAS5 is repressed by the YY1 transcription factor

**Figure 5.**
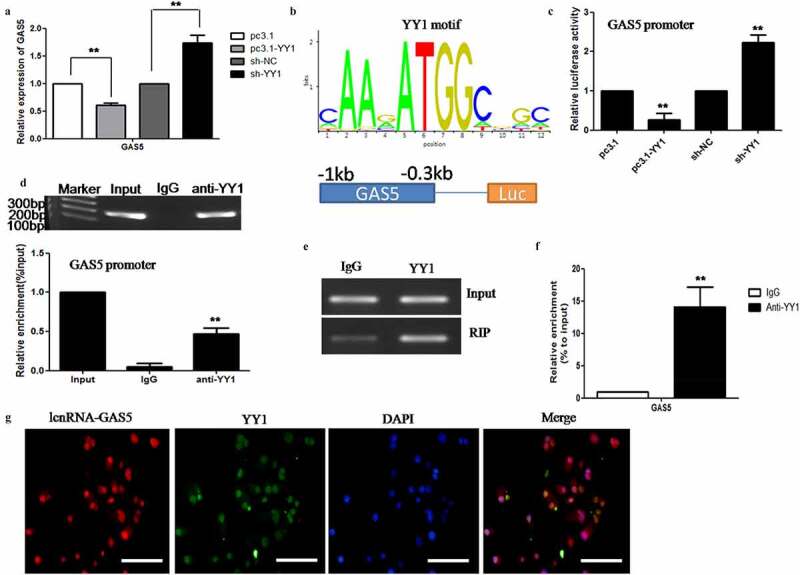
**LncRNA-GAS5 is represses by transcription factor YY1**. 5A: The pcDNA3.1 or pc3.1-GAS5 as well as sh-NC or sh-GAS5 plasmid were transfected respectively for 24 hours or 48 hours, the level of GAS5 expression level was detected by qPCR in HeLa cells. 5B: The binding sites of YY1 at GAS5 promoter was predicted by using online JASPAR software. 5C: Dual-luciferase reporter assays of GAS5 promoter activity in HeLa cells which were transfected with pcDNA3.1 or pc3.1-YY1 as well as sh-NC or sh-YY1 plasmids respectively. 5D: ChIP assay were performed to verify the binding between YY1 and the GAS5 promoter in HeLa cells. 5E-F: YY1 interacting with GAS5 in HeLa cells were detected by RIP assay. 5G: RNA-FISH detecting lncRNA-GAS5 (red) combined with immunofluorescence staining of YY1 (green) in HeLa cells. The nuclei were stained with DAPI (blue). Scale Bar: 10 μm

Many studies have shown that LncRNA regulates target molecules by interacting with transcription factors. We found that the knockdown of YY1 by sh-YY1 significantly increased the expression of GAS5 in HeLa cells, while the overexpression of YY1 inhibited the expression of GAS5 (Figure 5a). Next, we investigated whether YY1 suppresses GAS5 expression by regulating its transcription. We used an online transcription factor prediction software package, JASPAR (http://jaspar.binf.ku.dk/), to search for potential binding sites for YY1 within the promoter region of GAS5 (Figure 5b). Next, we constructed a luciferase reporter plasmid featuring the GAS5 promoter region and co-transfected this with pcDNA3.1-YY1 or sh-YY1 into HeLa cells. We found that YY1 inhibited the luciferase activity of the GAS5 promoter (Figure 5c). Furthermore, chromatin immunoprecipitation (ChIP) assays showed that YY1 bound to the promoter region of GAS5 (Figure 5d). To prove the interaction between YY1 and GAS5, we performed RNA immunoprecipitation (RIP) assays in HeLa cell lines. We demonstrated that GAS5 was enriched by anti-YY1 antibody, thus indicating that GAS5 interacted with YY1 directly (Figure 5e). RNA-FISH combined with immunofluorescence staining clearly demonstrated the colocalization of LncRNA-GAS5 and YY1 in the nucleus of HeLa cells (Figure 5f). These data indicate that YY1 inhibits the expression of GAS5 by inhibiting the transcription of the GAS5 promoter.

All data were presented as means ± SEM of three independent experiments, Values were significant at **p < 0.01 as indicated.

## *Acinetobacter baumannii* inhibited the expression of STX17 by relieving the inhibitory effect of YY1 on LncRNA-GAS5

**Figure 6.**
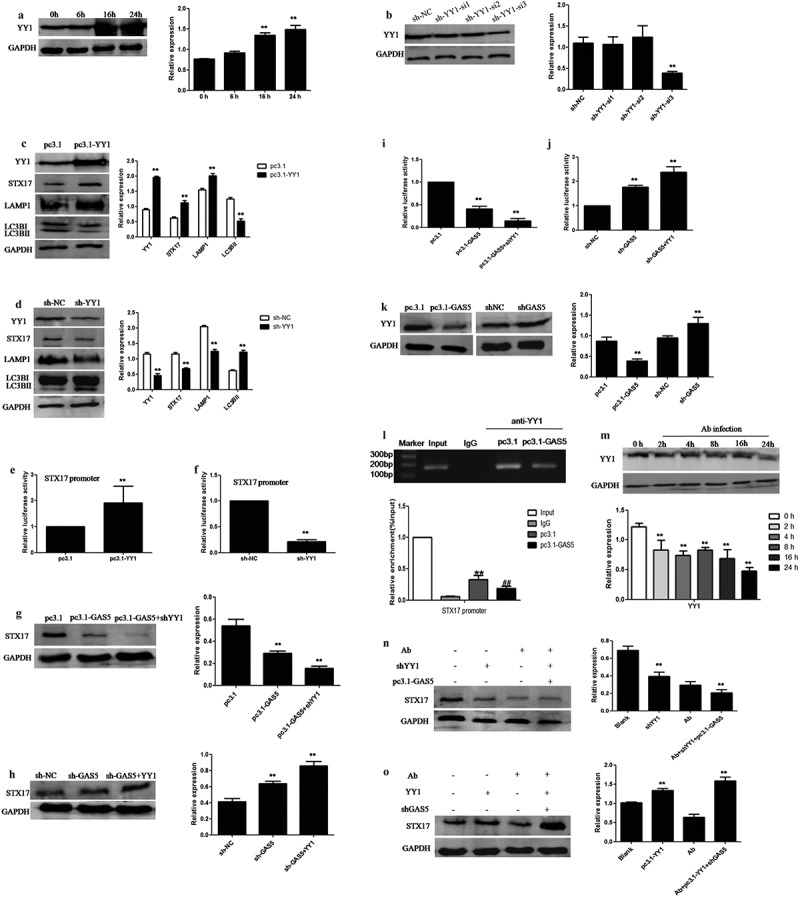
**LncRNA-GAS5 blocks STX17 expression by preventing YY1 from recruiting STX17 promoter in *Acinetobacter baumannii* infection**. 6A: HeLa cells were transfected with pc3.1-YY1 plasmid into HeLa cells for different times. The level of YY1 was assessed by Western blot assay. 6B: HeLa cells were transfected with sh-NC or sh-YY1 plasmid (sh-YY1-si1, sh-YY1-si2, sh-YY1-si3) into HeLa cells for 48 hours. The level of YY1 was assessed by Western blot assay. 6C: The expression level of autophagy related proteins in HeLa cells transfected with pcDNA3.1 or pc3.1-YY1 plasmid were detected by Western blot assay. 6D: The expression level of autophagy related proteins in HeLa cells transfected with sh-NC or sh-YY1 plasmid were detected by Western blot assay. 6E: Dual-luciferase reporter assays of STX17 promoter activity in HeLa cells which were transfected with pcDNA3.1 or pc3.1-YY1 plasmids respectively. 6F: Dual-luciferase reporter assays of STX17 promoter activity in HeLa cells which were transfected with sh-NC or sh-GAS5 plasmids respectively. 6G: HeLa cells were transfected with pcDNA3.1, pc3.1-GAS5 or co-transfection of pc3.1-GAS5 and sh-YY1 respectively, and then STX17 expression was detected by Western blot assay. 6H: HeLa cells were transfected with sh-NC, sh-GAS5 or co-transfection of sh-GAS5 and pc3.1-YY1 respectively, and then STX17 expression was detected by Western blot assay.6I: After transfected with pcDNA3.1, pc3.1-GAS5 or co-transfection of pc3.1-GAS5 and sh-YY1 respectively, the activity of STX17 promoter in HeLa cells were detected by Dual-luciferase reporter assay. 6J: After transfected with sh-NC, sh-GAS5 or co-transfection of sh-GAS5 and pc3.1-YY1, the activity of STX17 promoter in HeLa cells were detected by Dual-luciferase reporter assay. 6K: The expression of YY1 was evaluated by Western blot assay after transfected with pcDNA3.1 or pc3.1-GAS5 as well as sh-NC or sh-GAS5 plasmid respectively. 6L: The enrichment of YY1 at STX17 promoter region was evaluated via ChIP assay in HeLa cells transfected with pc3.1-GAS5 or pcDNA3.1. 6M: The expression of YY1 in HeLa cells infected with *Acinetobacter baumannii* for different times was detected by Western blot assay. 6N-O: Western blotting was used to detect the expression of STX17 in HeLa cells which the pc3.1-GAS5 and sh-YY1 plasmids or sh-GAS5 and YY1 plasmids co-transfected for 24 hours, and then infected with *Acinetobacter baumannii* for 24 hours

First, we investigated the efficacy of YY1 silencing and overexpression by transfecting with sh-YY1 and pcDNA3.1-YY1 and detecting expression levels by western blotting (Figure 6a-b). We also observed the changes in expression of STX17 and other autophagy proteins by overexpressing or knocking down YY1. We found that the overexpression of YY1 enhanced the expression of STX17 and LAMP1 but reduced the expression levels of LC3B. The knockdown of YY1 inhibited STX17 and LAMP1 expression but increased the expression of LC3B (Figure 6c-d). Similarly, Dual-luciferase reporter assays also found that the overexpression of YY1 increased the activity of the STX17 promoter while the knockdown of YY1 decreased the activity of the STX17 promoter (Figure 6e-f). Next, we examined the role of YY1 in the degradation of STX17 protein induced by GAS5 by western blotting and Dual-luciferase reporter assays. We found that the knockdown of YY1 further enhanced the inhibition of GAS5 on the expression of STX17 protein. However, the overexpression of YY1 further increased the expression of STX17 protein by GAS5 knockdown (Figure 6g-h). The luciferase activity of the STX17 promoter induced by the overexpression or knockdown of GAS5 could be reversed by the knockdown or overexpression of YY1 (Figure 6i-j). In addition, we also explored the effect of GAS5 on YY1 expression and found that the overexpression of GAS5 significantly inhibited the expression of YY1, while the knockdown of GAS5 significantly increased the expression of YY1, thus indicating that LncRNA-GAS5 acts in a feedback loop to inhibit YY1 (Figure 6k). Furthermore, we evaluated the role of GAS5 in the activation of the STX17 promoter by YY1 using chromatin immunoprecipitation assays and observed enrichment of YY1 to the endogenous STX17 promoter in HeLa cells. Moreover, the overexpression of GAS5 attenuated the binding of YY1 to the STX17 promoter (Figure 6l). Next, we investigated the role of GAS5 in the activation of STX17 protein by YY1 and found that GAS5 knockdown further enhanced the activation effect of YY1 on STX17 protein expression. However, the overexpression of GAS5 further attenuated the inhibition of STX17 protein by knocking down YY1 (Fig. S1A-B). Finally, we detected the effect of *Acinetobacter baumannii* on the expression of YY1 by western blotting and found that the expression of YY1 gradually decreased with the extension of infection time (Figure 6m). We also used western blotting to detect the effect of YY1 and GAS5 on STX17 reduction induced by *Acinetobacter baumannii* infection. The expression level of STX17 induced by *Acinetobacter baumannii* was lower in the case of GAS5 overexpression alone than in the case ofYY1 knockdown. In contrast, the effect of *Acinetobacter baumannii* on STX17 expression was higher when GAS5 was knocked down than when YY1 was overexpressed (Figure 6n-o). These experiments prove that the balance in expression of STX17 is regulated by YY1 and GAS5. More importantly, the degradation of STX17 induced by elevated GAS5 was regulated by YY1 in HeLa cells infected with *Acinetobacter baumannii*.

All data were presented as means ± SEM of three independent experiments, Values were significant at **p < 0.01 as indicated.

## The knockdown of GAS5 enhanced STX17 expression and attenuated *Acinetobacter baumannii* survival and inflammatory injury in the lung tissue *in vivo*

**Figure 7.**
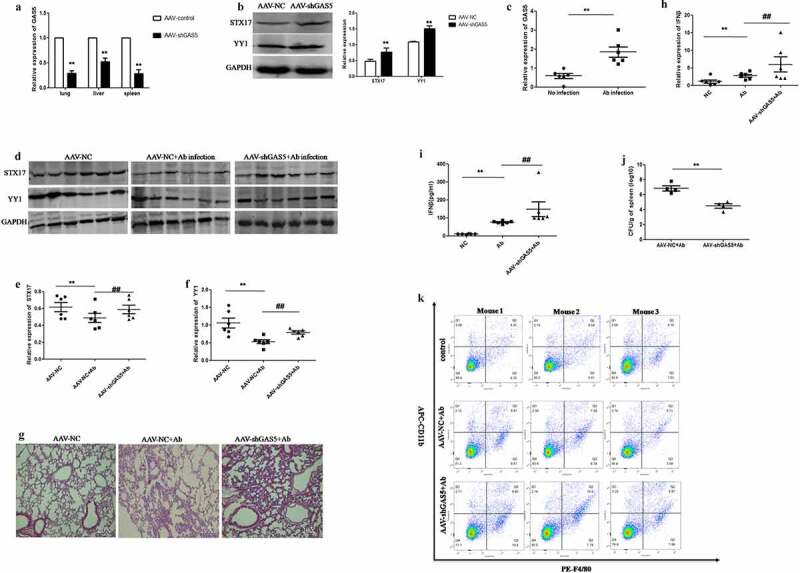
**Knockdown of GAS5 enhances STX17 expression and attenuates *Acinetobacter baumannii* survival and lung tissue inflammatory injury in vivo**Male C57/BL6 mice were transduced with AAV-NC or AAV-GAS5 virus via tracheal injection for three weeks, and then infected with *Acinetobacter baumannii* for 48 hours. 7A: The expression levels of GAS5 in lung, spleen and liver tissues were detected by qPCR (n = 6 per group). Data were presented as means ± SEM (n = 6 per group, **p < 0.01 compared with AAV-NC group). 7B: The expression of STX17 and YY1 in lung tissue were detected by Western blot assay. Data were presented as the means ± SEM (n = 6 per group, **p < 0.01 compared with AAV-NC group). 7C: The expression of GAS5 in lung tissue after infected with *Acinetobacter baumannii* at 48 h was detected by qPCR. Data are presented as means ± SEM (n = 6 per group **P < 0.01). 7D: The expression of STX17 and YY1 in lung tissue were detected by Western blot assay. Each lane presented an individual specimen. 7E-F: Statistical analysis of STX17 and YY1 expression in lung tissue of mice. GAPDH was used as an internal control. Data were presented as means ± SEM (n = 6 per group, **p < 0.01 compared with AAV-NC group, ##p < 0.01 compared with AAV-NC+ *Acinetobacter baumannii* group). 7G: Representative pictures of lungs tissues from WT mice, *Acinetobacter baumannii* infected mice, AAV-shGAS5 and *Acinetobacter baumannii* infected mice were shown. Lung tissues were subjected to Hematoxylin and eosin staining (n = 6 per group, Magnification ×20). 7H: The expression of IFN-β in lung tissue of mice was detected by qPCR. Data were presented as means ± SEM (n = 6 per group, **p < 0.01 compared with NC group, ##p < 0.01 compared with *Acinetobacter baumannii* infected group). 7I: The release of IFN-β in lung tissue of mice was detected by ELISA. Data were presented as means ± SEM (n = 6 per group, **p < 0.01 compared with NC group, ##p < 0.01 compared with *Acinetobacter baumannii* infected group). 7J: The number of *Acinetobacter baumannii* clones in lung tissue was detected by plate dilution counting method. Data were presented as means ± SEM (n = 4 per group, **P < 0.01 compared with AAV-NC + *Acinetobacter baumannii* infected group). 7K: The macrophages infiltration in spleens were analyzed by detecting CD11b and F4/80 by Flow cytometry, and the results obtained for three mice in each group were presented

Based on the results of our *in vitro* experiments, we decided to investigate the role of GAS5 in the lung injury of septic mice caused by *Acinetobacter baumannii* infection. We injected the adeno-associated virus carrying shGAS5 (named AAV-shGAS5) into the lungs of mice to generate GAS5 knockdown mice and then infected the mice with *Acinetobacter baumannii* for 48 hours. Firstly, we examined the knockdown efficiency of AAV-shGAS5 in different tissues from the experimental mice. We found that the expression levels of GAS5 in the lung, liver, and spleen, decreased significantly three weeks after the injection of AAV-shGAS5 (Figure 7a). In addition, western blot analysis confirmed that the expression of STX17 and YY1 were significantly increased in GAS5 knockdown mice (Figure 7b). Next, we found that the expression of GAS5 in the lung tissues of mice infected with *Acinetobacter baumannii* was significantly higher than that in the non-infected group, this was consistent with our cell experiments *in vitro* (Figure 7c). We also examined the role of GAS5 in the expression of STX17 and YY1 in the lung tissues of mice infected with *Acinetobacter baumannii* by western blotting. The expression of STX17 and YY1 in mice infected with *Acinetobacter baumannii* alone was significantly reduced while the expression of STX17 and YY1 in mice infected with *Acinetobacter baumannii* was significantly restored after the knockdown of GAS5 (Figure 7d-f). To explore the effect of GAS5 knockdown on lung injury induced by *Acinetobacter baumannii* infection in mice, we detected the degree of lung injury by Hematoxylin& Eosin (H&E) staining. The results showed that compared with the non-infected group, the lung tissue of mice infected with *Acinetobacter baumannii* showed inflammatory cell infiltration, the alveolar septum had widened and thickened, the size of the alveoli sac had decreased, and there was also evidence of bronchial hemorrhage. However, the degree of lung injury in mice infected with *Acinetobacter baumannii* but pretreated with AAV-shGAS5 was better than that in the *Acinetobacter baumannii* infection group (Figure 7g). In addition, we used qPCR and ELISA to detect the levels of IFN-β in the serum of GAS5 knockdown mice infected with *Acinetobacter baumannii* and found that the levels of IFN-β in the serum of GAS5 knockdown mice were significantly higher than those in mice that had only been infected with *Acinetobacter baumannii* (Figure 7h-i). The number of *Acinetobacter baumannii* in the lung tissue of GAS5 knockdown mice was significantly lower than that of non-knockdown mice (Figure 7j). Finally, we used flow cytometry to determine the number of macrophages in the spleen of mice infected with *Acinetobacter baumannii* after GAS5 knockdown. The results showed that GAS5 knockdown significantly increased the number of macrophages in the spleen induced by *Acinetobacter baumannii*. The mean number of macrophages was 4.99% in the control group, 6.86% in the *Acinetobacter baumannii* infection group, and 9.22% in the AAV-shGAS5 treatment group (Figure 7k). These results suggest that the knockdown of LncRNA-GAS5 can increase the expression of IFN-β to reduce the survival of *Acinetobacter baumannii* and improve the extent of lung injury.

## Discussion

In this study, we found that interactions between LncRNA-GAS5 and YY1 mediated the degradation of STX17 caused by *Acinetobacter baumannii*. The number of macrophages and the secretion of interferon-β increased in the lung tissue of mice lacking GAS5, thus enhancing the sensitivity against *Acinetobacter baumannii* infection. From a mechanistic point of view, *Acinetobacter baumannii* first reduced the expression of YY1, this indirectly led to the weakening of the inhibition of YY1 on GAS5 and the up-regulation of GAS5 expression. Subsequently, GAS5 directly activated the STX17 promoter to reduce its expression. In addition, the accumulation of GAS5 resulted in the detachment of YY1 from the STX17 promoter which indirectly reduced the expression of STX17.

LncRNA-GAS5 can destroy the autophagy degradation process, thus resulting in an imbalance in the host immune inflammatory response, this is not conducive for the host to fight against infection by *Acinetobacter baumannii*. The role of LncRNA in host bacteria interaction has been gradually revealed over recent years. A variety of pathogens, such as *tuberculosis, L. monocytogenes* and *Salmonella*, have been reported to activate or inhibit the transcription of different LncRNAs [29–31]. Although there are many reports relating to immune molecules in hosts against *Acinetobacter baumannii* infection, the role of LncRNA in this process has not been studied [32]. In this report, we demonstrated that the expression of GAS5 was elevated after *Acinetobacter baumannii* infection. Interestingly, we found that when the MOI value of *Acinetobacter baumannii* infection was 20, the expression of LncRNA-GAS5 began to decrease. This phenomenon indicated that an increase of infection concentration initiates a feedback regulation pathway in host cells, this leads to adaptive changes in the levels of LncRNA-GAS5 and may play a role in immune defense. However, the specific mechanisms underlying this phenomenon remain to be studied. Furthermore, the overexpression of GAS5 in HeLa cells led to a significant improvement in *Acinetobacter baumannii* infection. The knockdown of GAS5 resulted in a significant reduction in *Acinetobacter baumannii* infection. This suggests that the activation of GAS5 by *Acinetobacter baumannii* promotes its growth and replication. On the other hand, although GAS5 activates autophagy in HeLa cells, it can also inhibit the expression of the lysosomal marker LAMP1. Furthermore, GAS5 inhibits the expression of STX17, a key molecule that regulates the fusion of autophagosomes and lysosomes. Consequently, GAS5 can cut off the connection between autophagosomes and lysosomes to a certain extent, thus making it difficult for the host to remove *Acinetobacter baumannii*. Furthermore, *Acinetobacter baumannii* may use GAS5 to maintain its own replication and proliferation in autophagosomes, and minimize degradation by lysosomes, thereby avoiding an anti-bacterial immune response in the host. These findings demonstrate that GAS5 is an important molecule and regulates autophagy degradation to mediate host resistance to *Acinetobacter baumannii* infection.

**Figure 8.**
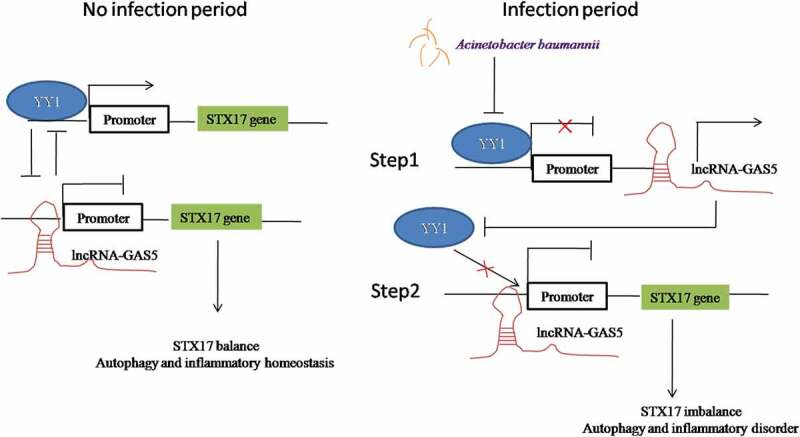
**A schematic model o*f Acinetobacter baumannii* regulating LncRNA-GAS5 and YY1 to inhibit STX17 expression**. Left panel was the resting state: LncRNA-GAS5 inhibited STX17 protein expression, while YY1 promoted STX17 protein expression. Because LncRNA-GAS5 and YY1 inhibited each other to keep the level of STX17 in balance, autophagy and inflammation were stable. Right panel was the *Acinetobacter baumannii* infection state: *Acinetobacter baumannii* inhibited the expression of transcription factor YY1, which relieved the inhibition of YY1 on LncRNA-GAS5. The abnormal expression of LncRNA-GAS5 resulted in the rapid decrease of STX17 protein. On the other hand, high level of LncRNA-GAS5 in turn enhanced the inhibition of YY1, resulting in the inactivation of the transcription of STX17 by YY1, thus aggravating the decrease of STX17 protein expression. Finally, this event leads to autophagy dysfunction and inflammation disorder

LncRNA regulates downstream gene expression in a variety of ways at the gene, transcription, and translation, levels. The subcellular localization of LncRNA determines its function. When located in the nucleus, LncRNA binds to transcription factors or RNA binding proteins (RBPs) to regulate the expression of various genes at the proximal or distal end [33]. By using FISH, we found that GAS5 was mainly localized in the nucleus, thus indicating that GAS5 plays a functional role in the multi-dimensional regulation of gene expression in the nucleus. We also investigated the interaction between GAS5 and the YY1 transcription factor. Zhang et al. reported that GAS5 could be suppressed by YY1 although GAS5 had no effect on YY1 [28]. This is not entirely consistent with our findings. In non-infected cells, YY1 inhibited the transcription of GAS5 and increased the expression of STX17. More importantly, YY1 was also targeted by GAS5 *via* a negative feedback loop. This regulatory mechanism maintains the balance of STX17 expression in cells, the negative feedback loop maintains stability in the host immune system. On the other hand, *Acinetobacter baumannii* was able to dynamically down-regulate the expression of YY1, thus releasing the suppression of YY1 on GAS5 by attenuating the suppressive effect of YY1 on the GAS5 promoter. In this way, the balance between YY1 and GAS5 was broken. The inhibitory effect of YY1 on GAS5 was weakened and the control of its expression was lost. In turn, activated GAS5 enhanced the inhibition of YY1. This exacerbated the downregulation of STX17 expression and led to disorder in the host’s autophagy system. These two pathways provided a mechanistic explanation for how *Acinetobacter baumannii* is able to down-regulate STX17 (Figure 8).

It is well known that type I IFN not only has an antiviral effect, but also plays an active role in a host’s resistance to bacterial infection. However, some bacteria can use their own virulence factors to control the release of type I IFN and disturb the immune inflammatory response [34,35]. In our study, we found that *Acinetobacter baumannii* activated macrophages in the spleen of mice and induced higher levels of IFN-β release than in the *Acinetobacter baumannii* infection group. Furthermore, the number of macrophages in the spleen, and the release of IFN-β in the serum of AAV-shGAS5 mice infected with *Acinetobacter baumannii*, also increased significantly. These effects further weaken the ability of *Acinetobacter baumannii* to survive and exert protective effects on tissue damage. However, the mechanism responsible for how LncRNA-GAS5 regulates the release of IFN-β mediated by *Acinetobacter baumannii* remains unclear and needs further investigation.

It is worth noting that Parra-Millán R et al. found that the transcription factor TFEB was activated by *Acinetobacter baumannii* and promoted the invasion of host cells [36]. TFEB plays a key role in the regulation of lysosomal biogenesis. The activation of TFEB by *Acinetobacter baumannii* leads to the production of autophagosome and lysosomal markers in large quantities. This behavior seems to be more beneficial for the host to eliminate infection, but is not conducive to the survival of the bacteria. We believe that the real reason for the enhanced invasiveness of *Acinetobacter baumannii* after the activation of TFEB does not lie in changes in the amount of autophagosome and lysosome markers, but in terms of whether it interferes with the degradation function of autolysosomes. In the future, we should include TFEB to further analyze the destructive effect of *Acinetobacter baumannii* on autophagy degradation.

In summary, we investigated the mechanism by which LncRNA-GAS5 interacts with the YY1 transcription factor to regulate STX17 in the autophagy degradation disorder induced by *Acinetobacter baumannii* infection. Our findings provide a potential therapeutic target for the treatment of inflammatory injury caused by *Acinetobacter baumannii* infection.

## Supplementary Material

Supplemental Material

## Data Availability

The data that support the findings of this study are available from the corresponding author upon reasonable request.
