# Supplementary material for: Acinetobacter baumannii up-regulates LncRNA-GAS5 and promotes the degradation of STX17 by blocking the activation of YY1
Source: Virulence. 2021 Jul 25;12(1):1965–79. doi: 10.1080/21505594.2021.1953851 (PMC8312602; doi:10.1080/21505594.2021.1953851)
Supplement: Supplemental Material [file KVIR_A_1953851_SM7857.zip › Table_S1.docx]

| **Gene** | **Primer** | **Sequence(5′-3′)** |
| --- | --- | --- |
| **qPCR**  **Human primers**  GAS5  STX17  GAPDH  **Mouse primers**  GAS5  STX17  IFN-β  Actin  **CHIP assay**  GAS5 promoter  STX17 promoter  **RNA interference**  STX17-si-1  STX17-si-2  STX17-si-3  GAS5-si-1  GAS5-si-2  GAS5-si-3  YY1-si-1  YY1-si-2  YY1-si-3 | forward  reverse  forward  reverse  forward  reverse  forward  reverse  forward  reverse  forward  reverse  forward  reverse  forward  reverse  forward  reverse  forward  forward  forward  forward  forward  forward  forward  forward  forward | TAATGACCACAACAAGCAAGC  TTAAAATTGGAGACACTGTT  GAGAAATTGAGAAACTTTGTTTG  AATGGAGTTGGAGAAATTCTGC  GGAGCGAGATCCCTCCAAAAT  GGCTGTTGTCATACTTCTCATGG  TGTGGACCTCTGTGATGGGA  ACATTGCGCTCGCTCTGTTA  CTAGGCGGGAGGTGTTTCTG  AGCCTGCGTAACTTCACCTT  TGGGAGATGTCCTCAACTGC  CCTGCAACCACCACTCATTC  CATTGCTGACAGGATGCAGAAGG  TGCTGGAAGGTGGACAGTGAGG  TTACTTCCCGGATGGTTTCCA  AGTCCTTCCCCCTACCTTCTG  AACGGACAGGAAAACTGCGA  GCTGCTGAGAACAGCGAAAG  GAUAGAUCCUGUUAAAGAAdTdT  GCAAUUUAAUGAUGAAGAAdTdT  GACAAGAAAUGCAGUUAAAdTdT  GGCUCUGGAUAGCACCUUA  GGAUGAGAAUAGCUACUGA  GACCUGUUAUCCUAAACUA  CGACGACTACATTGAACAA  GATGATGCTCCAAGAACAA  AGAAGCAGGTGCAGATCA |
